# Distribution of methane-cycling archaea in buried ridge flank sediment: community zonation, activity, and potential environmental drivers

**DOI:** 10.3389/fmicb.2025.1710699

**Published:** 2025-11-24

**Authors:** Mark Alexander Lever, Yuki Morono, Fumio Inagaki, Andreas P. Teske

**Affiliations:** 1 Department of Marine Science, Marine Science Institute, University of Texas at Austin, Port Aransas, TX, United States; 2 Earth, Marine and Environmental Sciences, University of North Carolina at Chapel Hill, Chapel Hill, NC, United States; 3 Kochi Institute for Core Sample Research, Japan Agency for Marine-Earth Science and Technology (JAMSTEC), Kochi, Japan; 4 Advanced Institute for Marine Ecosystem Change (WPI-AIMEC), JAMSTEC, Yokohama, Japan; 5 WPI-AIMEC, Tohoku University, Sendai, Japan

**Keywords:** deep biosphere, subseafloor sediment, methanogenesis, methanogens, anaerobic oxidation of methane, ANaerobic MEthane oxidizing archaea, metal cycling, methyl coenzyme M reductase

## Abstract

Subseafloor sediments harbor Earth’s biggest reservoir of methane, with most of this methane being produced biologically by methanogenic archaea (methanogens). Yet, little is known about the controls on *in situ* abundances, community structure, and biochemical pathways of methanogens, and closely related anaerobic methane oxidizing archaea (ANMEs), in these environments. Here we examine the vertical distribution of methane-cycling archaea at Integrated Ocean Drilling Program Site U1301, an offshore drilling site on the eastern flank of the Juan de Fuca Ridge, that was previously characterized with respect to its *in situ* temperature profile, sediment provenance, geochemical gradients, and general microbial community structure. We integrate (1) functional gene analyses (*mcr*A) to analyze methane-cycling archaeal abundances and community structure with (2) sediment porewater concentration profiles of sulfate, methane, Mn^2+^, and Fe^2+^ to shed light on the distributions of dominant microorganisms and processes involved in subseafloor methane cycling. These analyses indicate that sediments from the cold seafloor (2 °C) to the hot basement (64 °C), across zones of sulfate reduction (SR), anaerobic oxidation of methane (AOM), and methanogenesis (MG), were dominated by two phylogenetic clusters. These belonged to the family *Methanoperedenaceae*, which couples the oxidation of methane to the reduction of nitrate or metals, and the candidate order Methanophagales (group ANME-1a-b). The latter has mainly been linked to sulfate-dependent AOM, although a facultative methanogenic metabolism has also been proposed. Other groups of methane-cycling archaea (ANME-2c, -3, *Methanothrix*, *Methanocellales*, *Candidatus* Nezhaarchaeales) were only detected in single samples from the upper and lower AOM zones and the MG zone. No stimulation of methanogenic activity was evident at the deep sediment-basement interface; however, the dominant *Methanoperedenaceae* and Methanophagales phylotypes throughout the sediment column cluster phylogenetically with those previously detected in underlying basalt, suggesting dispersal and similar selective forces on methane-cycling archaea across this major lithological boundary. Based on the observed dominance of *Methanoperedenaceae* and porewater geochemical profiles which indicate decoupling between measured sulfate and methane concentrations in sediments with methane oxidation, we propose that a significant portion of AOM in the iron- and manganese-rich sediments of the Juan de Fuca Ridge Flank proceeds through the reduction of iron (III) and manganese (IV).

## Introduction

Despite hosting a significant percentage of global microbial biomass (Kallmeyer et al., 2012; Parkes et al., 2014), subseafloor sediments are among the least understood ecosystems on Earth. Research based on sediment cores recovered by ocean drilling has demonstrated the presence of intact and metabolically active cells to >2,000 m below the seafloor (mbsf) (Inagaki et al., 2015) at temperatures ≥100 °C (Roussel et al., 2008; Heuer et al., 2020). The primary energy source of these microorganisms is ancient organic matter. This organic matter continues to be broken down, even millions of years after its initial burial at the seafloor (D’Hondt et al., 2019), albeit at very low rates that supply only minimal amounts of power to surviving cells (1.50 × 10^−20^ to 2.23 × 10^−18^ watts per cell; Bradley et al., 2020). While aerobic organic matter mineralization dominates large portions of abyssal subseafloor sediment (D’Hondt et al., 2015), the increased sedimentary input of organic matter in continental shelf and slope environments typically leads to O_2_ depletion in the uppermost centimeters or decimeters (Jørgensen and Kasten, 2006). Below this oxic surface layer, microbial organic matter mineralization proceeds via fermentative processes that are coupled to anaerobic respiration reactions involving the reduction of nitrate, manganese oxides, iron oxides, and sulfate (Froelich et al., 1979; D’Hondt et al., 2004). Among the latter, sulfate reduction (SR) is quantitatively the most important reaction in marine sediments (Bowles et al., 2014; Jørgensen et al., 2024).

Archaeal methane production (hereafter referred to as ‘methanogenesis) is another widespread microbial process in anoxic sediments and mainly proceeds through three pathways: (1) the reduction of CO_2_ (or formate) to methane, (2) the disproportionation (dismutation) of acetate to methane and CO_2_, and (3) the disproportionation or reduction of C1 compounds (methanol) or methyl groups (e.g., methyl amines, and methyl sulfides) to methane and CO_2_ (Whitman et al., 2014; Kröninger et al., 2017). While methanogenesis co-occurs at low rates with microbial nitrate, manganese, iron, and sulfate reduction, it typically only becomes dominant in deeper layers where these electron acceptors have been depleted (methanogenesis (MG) zones). This is because electron acceptors, such as nitrate, metal oxides, and sulfate provide higher *in situ* energy yields than methanogenesis from key fermentation products, such as H_2_, formate, or acetate (e.g., Hoehler et al., 1998). An exception to this rule are methanol and methylated substrates, so-called ‘non-competitive substrates’, that are not consumed by most sulfate and metal reducers and support low rates of methanogenesis in the presence of sulfate and metal oxides. Even though most methane production is restricted to deep, highly energy-depleted MG zones, the slow rates of methane production and methane accumulation in these layers over geologic time scales explain why marine sediments are Earth’s biggest methane reservoir (Wallmann et al., 2012). Most sedimentary methane is produced by highly specialized members of the archaeal kingdoms Methanobacteriati (formerly Euryarchaeota) and Thermoproteota (formerly TACK (Thaumarchaeota-Aigarchaeota-Crenarchaeota-Korarchaeota)) that are widely referred to as ‘methanogens’. Among these, the methanogenic Methanobacteriati are far more studied and physiologically characterized, and have also been detected more frequently in marine sedimentary environments, than the more recently discovered methanogenic Thermoproteota (Wen et al., 2017).

Even though episodic eruptions of subseafloor methane to the atmosphere have been linked to major climatic shifts in Earth’s past (Kim and Zhang, 2022), the spatially and quantitatively vast methane reservoir in marine sediments only contributes a minor percentage of global atmospheric methane emissions today (Dean et al., 2018). This is because marine methane emissions are kept low by ANaerobic MEthane oxidizing archaea (ANMEs), close relatives of methanogens, that oxidize methane via a reversal of the canonical methanogenesis pathway. ANMEs occur predominantly in sedimentary horizons on the edge of MG zones, where diffusion results in the co-occurrence of significant sulfate and methane concentrations (Reeburgh, 2007). ANMEs living in these so-called ‘sulfate–methane transitions’ mediate the anaerobic oxidation of methane (AOM) coupled to SR, with the latter in many cases being performed by syntrophic sulfate-reducing bacteria. This sulfate-dependent AOM, as well as other forms of AOM that are coupled to nitrate, manganese, or iron reduction (Haroon et al., 2013; Ettwig et al., 2016; Leu et al., 2020), also occur outside of AOM zones, and may, in addition to the low methane production rates, explain why methane accumulation is usually very low (micromolar) outside of MG zones (Deng et al., 2025).

The drivers of community structure and metabolic pathways of methanogens and ANMEs in subseafloor sediments remain poorly understood. There have only been few methanogenic isolates from subseafloor sediments (e.g., *Methanoculleus submarinus*; Mikucki et al., 2003). Moreover, methane-cycling archaea are often rare or even absent from subseafloor DNA sequencing surveys targeting universal prokaryotic or archaeal 16S ribosomal RNA genes, or metagenomes—even in sediments with elevated concentrations of biogenic methane (e.g., Parkes et al., 2005; Torres et al., 2015; Bojanova et al., 2023). This has resulted in a surprisingly small and patchy data set on subseafloor methane-cycling archaea and the proposition that these archaea only represent low percentages (<1%) of the total microbial community (Lever, 2013). PCR assays that specifically target methane-cycling archaea have been more successful than universal 16S rRNA or metagenomic assays. Herein the main focus has been on *mcr*A, a functional gene encoding the alpha subunit of methyl coenzyme M reductase, which is found in all methane-cycling archaea. The targeting of *mcr*A has resulted in the first detailed community profile of methane-cycling archaea in deep subseafloor sediments (0–230 mbsf) of the Peru Trench (Ocean Drilling Program (ODP) Site 1,230; Lever et al., 2023). This community profile indicated (near) absence of methane-cycling archaeal populations in sulfate-rich sediments, but the presence of putatively methanotrophic ANME-1a-b (order Methanophagales) throughout the AOM zone and of putatively aceticlastic methanogenic *Methanotrichaceae* and ANME-1d archaea throughout the MG zone (Lever et al., 2023). Based on observed environmental distributions, ANME-1d were proposed to be methanogenic (Lever et al., 2023; Deng et al., 2025).

The study of the Peru Trench (Lever et al., 2023) reaffirmed the importance of sulfate in driving methane-cycling archaeal community structure, while also suggesting that growth of these archaea occurs in these sediments once sulfate is depleted. Yet, the high physicochemical homogeneity of ODP Site 1,230 (D’Hondt et al., 2003; Lever et al., 2023) precluded an assessment of how other factors, such as temperature, organic matter content, organic matter sources, or lithology might influence methane-cycling archaea in subseafloor sediments. Here we examined the vertical distribution of methane-cycling archaea at Integrated Ocean Drilling Program (IODP) Site U1301, on the eastern flank of the Juan de Fuca Ridge in the northeastern Pacific. Our main goal was to shed light onto the environmental controls on methanogenic and methanotrophic communities and activities in ridge flank sediments. Site U1301 is well-suited for this investigation as its sediment column covers a range of variables that are considered important drivers of subseafloor life. These include sediment age [0 to ~1.7 million years (Ma)], temperature (2 to 64°C), lithology (hemipelagic marine sediments, terrestrial turbidite deposits, volcanic ash, sediment-basement interface), and geochemistry (e.g., a deep MG zone that is above and below flanked by AOM and SR zones). Despite accumulation of biologically produced methane to millimolar concentrations in the MG zone, no DNA sequences of known methane-cycling archaea were recovered during previous sequencing efforts of U1301 sediments based on 16S ribosomal RNA genes and metagenomes (Labonté et al., 2017; Metze et al., 2023). However, previous research targeting *mcr*A has resulted in the successful detection of methane-cycling archaea in the underlying basaltic basement of U1301 (Lever et al., 2013).

In this study, we first determine the vertical distributions of microbial MG, SR, and AOM based on measured porewater concentrations of methane and sulfate. We then compare *mcr*A abundances and sequence distributions to inferred process distributions, as well as to published data on *in situ* temperature, lithology, organic matter sources, carbon stable isotopes, and dissolved metal (Mn^2+^, Fe^2+^) concentrations to determine potential drivers of methane-cycling archaeal activity and community structure. Despite major changes in temperatures, lithologies, and geochemical conditions, the same two groups of methane-cycling archaea, belonging to the family *Methanoperedenaceae* and ANME-1a-b group, dominate the U1301 sediment column. These same two groups also dominate underlying basalt at U1301 (Lever et al., 2013), suggesting potential for microbial dispersal and colonization across the deep sediment-basalt interface.

## Materials and methods

### Study site

**Figure 1:**
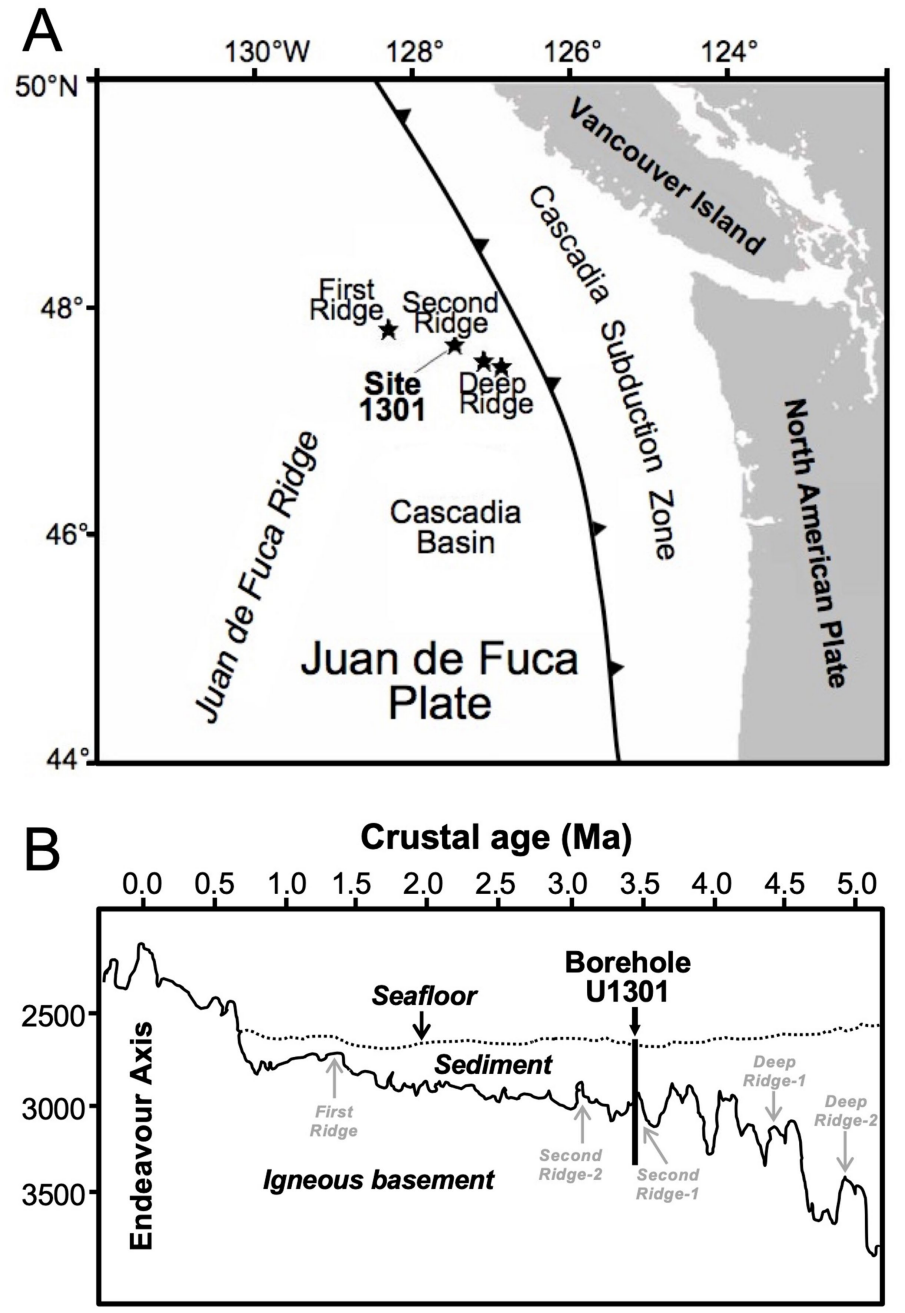
**(A)** Map of study region, showing location of IODP Site U1301. **(B)** Cross-sectional view of the eastern flank of the Juan de Fuca Ridge, from the Endeavour Segment in the west (northern part of ridge) to the subduction zone in the east. The distance from U1301 to the Endeavour Axis is ~100 km. **(A)** and **(B)** are modified from Fisher et al. (2005).

In summer 2004, using the drilling vessel JOIDES Resolution, IODP Expedition 301 sampled subseafloor sediment core samples at Site U1301 on the Juan de Fuca Ridge Flank at a water depth of 2,656 m, ~400 km west of the coast of Oregon, USA (Figure 1). This site is well-suited for the study of subsurface microbiology due to well-characterized sediment history, lithology, temperature regimes, as well as geochemical gradients that indicate microbial activity throughout the sediment column (Davis et al., 1997; Fisher et al., 2005). Lithostratigraphic analyses indicate a 265-m thick, lithologically heterogeneous sediment column that can be divided into two stratigraphic units (I and II; Fisher et al., 2005). Unit I (0-215mbsf) is dominated by turbidite sediments with significant terrestrial contributions that include volcanic ash from the Cascade Range and Columbia River basalts. The upper interval, Subunit IA (0–13.1mbsf), contains thinly bedded layers of silty and fine-grained sandy turbidites and hemipelagic clay, whereas the lower interval (Subunit IB; 13.1-215mbsf) is dominated by thinly bedded to massive bedded, fine- to coarse-grained turbidites (Fisher et al., 2005). Unit II (215–265 mbsf) consists almost entirely of hemipelagic clay. Based on magnetic abnormalities, the underlying basaltic basement is estimated to be 3.5 Ma (Davis et al., 1997). Nannofossil analyses indicate that sediment accumulation began considerably later (~1.7 Ma; Davis et al., 1997; average sedimentation rate: 156 mm ka^−1^).

Though thick layers of sediment prevent significant vertical advection, diffusion-mediated exchanges profoundly influence the biogeochemical zonation throughout the sediment column (Wheat and Mottl, 1994). Sulfate diffuses into sediment from both overlying seawater and the underlying basaltic aquifer, creating a bimodal sulfate distribution, where only a ~ 65-m depth interval in the middle of the 265-m thick sediment column is sulfate-depleted due to *in situ* microbial SR (Fisher et al., 2005). This interval contains an MG zone, in which microbially produced methane concentrations reach values of >5 mM. The considerably high microbial activity at U1301 contrasts with the low organic carbon content, which drops from ~0.91 dry weight percent (wt. %) at 1.5 mbsf to values around 0.3 wt. % (range: 0.07 to 0.7 wt. %) below 10 mbsf. Herein, values below 0.1 wt. % fall into terrestrial turbidites, in which C:N ratios of 20 to 37 indicate predominance of terrestrial plant-derived organic matter (Fisher et al., 2005).

Sediment temperatures increase linearly with depth, from ~2 °C at the seafloor to 64°C at the basement (Fisher et al., 2005). Though these temperatures remain well within the known temperature limits of life, heating of sediments from the hydrothermally active basement may influence microbial activity and community structure, due to differences in temperature ranges among microbes, and potentially by producing microbial energy substrates via the thermal breakdown of recalcitrant organic matter. In addition, increases in microbial maintenance energy requirements due to temperature-driven increases in biomolecule decay rates (e.g., racemization, hydrolysis) could affect microbial community structure and population size with increasing sediment depth (Lever et al., 2015).

### Sampling

Using advanced piston coring, high quality sediment cores were obtained from two boreholes (Holes U1301C and U1301D) (Fisher et al., 2005). U1301D was offset by 18 m from U1301C and was used to recover sediments from 120 to 180 mbsf that were not cored in U1301C. Cores used for porewater geochemical analyses and DNA extractions were sectioned right after arrival on deck. Sediments for DNA analyses were frozen at −80 °C immediately after core sectioning. Contamination of drilling fluid was measured in the same core sections, in samples adjacent to ones used for molecular biological and geochemical analyses, by measuring concentrations of perfluoromethylcyclohexane, a chemical tracer with which drilling fluid had been amended to a concentration of 1 mg L^−1^ (Lever et al., 2006). For molecular biological analyses, we only used the effectively contamination-free core centers (Lever et al., 2006).

### DNA extraction

Aliquots from core centers (6 g) were homogenized in 50-ml Falcon tubes with artificial seawater [10 ml; contained 3% (*w/v*) sodium chloride (NaCl), 0.3% magnesium sulfate (MgSO_4_), 0.2% potassium chloride (KCl), 20 mM (0.312 g) sodium monophosphate (NaH_2_PO_4_)], incubated at room temperature for 2 h, and then centrifuged (20 min, 4,000 × *g*). Supernatants containing extracellular DNA were discarded, whereas sediment pellets, containing whole cells, were kept. A modification of the ISOIL Large for Beads kit (Nippon Gene, Tokyo, Japan), that included a proteinase K incubation step, was used to extract DNA. Following this protocol, sediment pellets were added to Bead Tubes along with 9.5 ml Lysis Solution BB, and 0.5 ml Lysis Solution 20 S. Contents were homogenized by vortexing, and subsequently shaken to mechanically break cells (30s, room temperature) using a ShakeMaster (BioMedical Science, Tokyo, Japan). We then inserted a step in which PCR-inhibitory proteins were removed by proteinase K addition (500 μg ml^−1^) and gentle rotation on a shaker table (50 °C, 2 h). The temperature was then increased to denature the proteinase (65 °C, 1 h). From then on, the ISOIL extraction method was followed according to protocol. The final DNA extract was sequentially purified and concentrated using the (1) Amicon Ultra-15 10 K kit, (2) Montage PCR Cleanup kit (both Millipore Corporation, Billerica, USA), and (3) Mag Extractor—PCR & Gel Clean Up kit (Toyobo, Tokyo, Japan). All samples were extracted at the Kochi Institute for Core Sample Research of JAMSTEC (Kochi, Japan), except one sample from 1301D (Core 11H-1; 91.7 mbsf), which was extracted at the Marine Science Department of the University of North Carolina at Chapel Hill (Chapel Hill, USA).

### PCR amplification

All known methane-cycling archaea use the reductive acetyl CoA pathway to produce or oxidize methane. The gene for the alpha subunit of methyl coenzyme M reductase (*mcr*A), an enzyme that is reduced by coenzyme B during the terminal step of MG (Thauer, 2019), is a widely used phylogenetic marker for methane-cycling archaea due to its high degree of sequence conservation and being present in all known methanogens and ANMEs (Friedrich, 2005). In anaerobic methanotrophs, which perform a reversal of the methanogenic pathway, *mcr*A is involved in the oxidation of coenzyme B during the initial step of the reaction (Thauer, 2019).

*Mcr*A was below detection when PCR-amplified with published mcrI (Springer et al., 1995) and ME1/ME2 primers (Hales et al., 1996), but amplified successfully with the mcrIRD primer pair (F: 5’-TWY GAC CAR ATM TGG YT; R: 5’-ACR TTC ATB GCR TAR TT), a revision of the mcrI primer pair, that uses fewer degeneracies without compromising phylogenetic breadth (Lever and Teske, 2015). Since the mcrIRD primer pair does not amplify *mcr*A sequences of Methanophagales (ANME-1), we used the group-specific ANME-1-mcrI primer pair to target *mcr*A genes belonging to this group (F: 5′-GAC CAG TTG TGG TTC GGA AC; R: 5′-ATC TCG AAT GGC ATT CCC TC; Lever and Teske, 2015).

We used the TaKaRa SpeedStar Polymerase Kit for PCR amplifications (TaKaRa Bio Inc., Shiga, Japan). The PCR reaction mix was as suggested in the manual, except that we doubled the Taq concentrations, and included bovine serum albumin to a concentration of 4 *μ*g per μL of the reaction mix. The PCR protocol consisted of (1) 1 × 2 min denaturation (98 °C), (2) 40 × (a) 30s denaturation (95 °C), (b) 30s annealing (mcrIRD: 55°C; ANME-1-mcrI: 63 °C), (c) 1 min extension (72 °C), and (3) 1 × 5 min extension (72 °C). PCRs were performed using Veriti model thermal cyclers (Applied Biosystems, Foster City, USA).

Since non-target DNA bands occurred occasionally in PCR products obtained with mcrIRD primer pair that were inspected by gel electrophoresis, reliable quantification of gene copies by real-time PCR assays was not possible. Instead, we quantified *mcr*A copy numbers per gram sediment by most-probable-number (MPN) PCR assays. Dilution series consisted of 10 μL, 3 μL, 1 μL, 0.3 μL, 0.1 μL of DNA extract per 25-μL reaction volumes. The resulting PCR products were checked by gel electrophoresis. Then, the minimum number of *mcr*A copies per volume of extract was determined based on the most diluted DNA extract that generated PCR amplicons in the known size range of *mcr*A. Minimum *mcr*A copy numbers per gram of sediment were then calculated and converted to minimum copy numbers per volume of sediment by multiplication with measured sediment wet densities, the latter obtained from the same whole-round core samples as DNA samples (Fisher et al., 2005). In agreement with previously published results, which reported one *mcr*A copy per methane-cycling archaeal genome (Reeve et al., 1997), we assumed *mcr*A copy numbers to equal cell numbers per volume of sediment.

### Cloning and sequencing

PCR products were purified with the Montage PCR Cleanup kit (Millipore Corp., Billerica, USA), cloned and inserted into chemically competent *E. coli* using the Topo TA Kit following the manufacturer’s instructions (Invitrogen, Carlsbad, USA), and sequenced via the Sanger method as previously described (Inagaki et al., 2004). Per primer combination and sample, 48 colonies were picked for sequencing. The only exceptions were PCR products obtained with mcrIRD F/R from Core 7H-2 and Core 11H-1 (both Borehole C), from which 96 clones were picked for sequencing.

### Phylogenetic analyses

Phylogenetic assignments of *mcr*A reads were performed in ARB (Ludwig et al., 2004; www.arb-home.de) using neighbor-joining phylogenetic trees with Jukes Cantor correction and 1,000 bootstrap replicate calculations. Trees were constructed using a public database (mcrA4All; Lever et al., 2023) with >2,400 high-quality, optimally aligned *mcr*A sequences from pure cultures, as well as amplicon sequencing, metagenome, and whole-genome studies. To explore sequence similarities among *Methanoperedenaceae* and ANME-1a-b *mcr*A sequence clusters from U1301 sediment and basalt core samples, the percent nucleotide differences were calculated via pairwise comparisons using the Maximum Composite Likelihood model in MEGA12 (Stecher et al., 2020).

### Nucleotide sequence accession numbers

All *mcr*A phylotype sequences are publicly accessible through the National Center for Biotechnology (NCBI) homepage (nucleotide accession numbers PX095117-PX095131).

## Results

### Vertical distributions of SR, AOM, and MG

**Figure 2:**
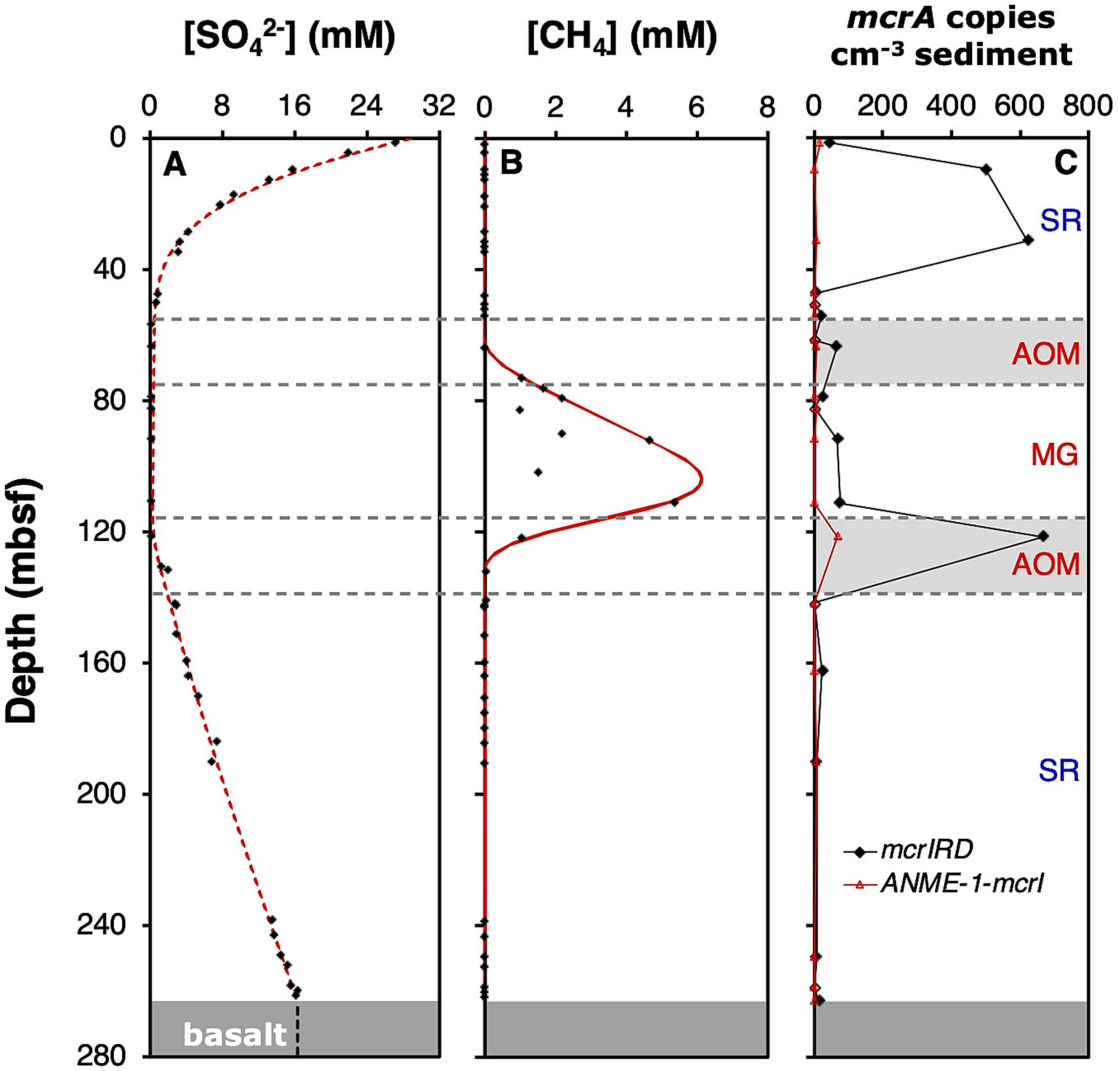
Depth profiles of **(A)** sulfate concentrations; **(B)** methane concentrations; and **(C)** *mcrA* copy numbers based on MPN-PCR using the mcrIRD and ANME-1-mcrI primer pairs (grey shaded areas = AOM zones). Red lines in **(A,B)** are best fit lines. Samples with low methane concentrations in the MG zone were assumed to have experienced significant outgassing during core recovery and sampling. Sulfate and methane concentrations were previously published in Fisher et al. (2005).

Porewater concentration profiles of methane and sulfate indicate a vertically structured microbial activity profile. In the upper sediment column (0–65 mbsf), consumption of seawater-derived sulfate by sulfate reducing microorganisms results in a concave-up profile (Figure 2A) with peak rates of SR near the seafloor. Sulfate is consistently depleted from ~65–120 mbsf and increases nearly linearly from 120 mbsf to the basement, suggesting significant SR rates between ~115–140 mbsf and near zero SR rates below. Dissolved methane concentrations remain between 1 and 2 μM from the seafloor to 65 mbsf (Figure 2B). Concurrent with the depletion of sulfate, methane concentrations increase in the interval from 65 to 130 mbsf, reaching millimolar concentrations from 73 to 121 mbsf. Dissolved methane concentrations in the lower SR zone are slightly higher than in the upper SR zone (2–4 μM). Virtually all methane produced in the MG zone is consumed in adjacent sediment layers by AOM (Figure 2B). The upper AOM zone ranges from ~55 to 75 mbsf, whereas the lower AOM zone extends from ~115 to 140 mbsf. Notably, measured sulfate concentrations near analytical background indicate minimal SR rates within the upper AOM zone, in spite of significant methane consumption by AOM.

### McrA abundance, distribution, and diversity

*Mcr*A was detected in 15/19 core samples from the sediment column of U1301, indicating the presence of methane-cycling archaea throughout the SR, AOM, and MG zones (Table 1). PCR with serially diluted DNA extracts using the general mcrIRD primer pair demonstrate two peaks in *mcr*A copy numbers (both ≥500 copies cm^−3^ sediment), one near the seafloor in the upper SR zone (9.4–31.3 mbsf), and one in the lower AOM zone (121.5 msf; Figure 2C; Table 1). Additional, smaller peaks (≥50 copies cm^−3^) occurred in the upper AOM zone and lower half of the MG zone, whereas comparatively lower *mcr*A copy numbers were detected in deeper layers of the upper SR zone and throughout the lower SR zone (Figure 2D; Table 1). *Mcr*A was below detection of the mcrIRD primer pair in 5/19 core samples throughout the sediment column, showing no clear trend in relation to depth, temperature, lithology or biogeochemical zone (Table 1). By comparison, the ANME-1-mcrI primer pair only detected *mcr*A in 6/19 samples. These samples were distributed throughout both SR and AOM zones and the MG zone. The highest minimum copy numbers were in the lower AOM zone (≥67 copies cm^−3^), and thus roughly 10fold lower than the highest minimum copy numbers obtained with the mcrIRD primer (Table 1).

**Table 1:** Overview of Borehole and Core IDs, sampling depths, extrapolated *in situ* temperatures, lithological subunits, dominant sediment grain size, and biogeochemical zone in relation to *mcr*A distributions based on two primer combinations (mcrIRD, ANME-mcrI).

Bore-hole	Core ID^1^	Depth (mbsf)	T (°C)	Sub-unit	Litho-logy^2^	Biogeo-chemical zone^3^	mcrIRD^4^		ANME-1-mcrI^5^		Total^6^
							Minimum copies cm^−3^	Groups detected (# of clones)	Minimum copies cm^−3^	Groups detected (# of clones)	Minimum copies cm^−3^
C	1H-2	1.5	2.2	IA	clay	SR	46	Mperedens (4)	15	ANME-1a, -c, -d (43)	61
C	2H-3	9.4	4.1	IA	clay	SR	498	Mperedens (37)	bd	bd	498
C	4H-5	31.3	9.3	IB	fine sand	SR	623	Mperedens (40)	6	ANME-1-d (45)	629
C	6H-3	47.2	13.1	IB	sand	SR	7	Mperedens (44)	bd	bd	7
C	6H-6	50.6	13.9	IB	clay	SR	bd	bd	bd	bd	bd
C	7H-2	54.1	14.7	IB	clay	SR	20	Mperedens (73)	bd	bd	20
C	7H-7	61.6	16.5	IB	fine sand	AOM	bd	bd	bd	bd	bd
C	8H-2	63.6	17.0	IB	fine sand	AOM	65	Mperedens (44)	7	ANME-1-b (45), Nezhaar (1)	72
C	9H-5	78.7	20.5	IB	clay	MG	22	Mperedens (14)	bd	bd	22
C	10H-1	82.2	21.4	IB	sand	MG	bd	bd	6	ANME-1-d (29)	6
C	11H-1	91.7	23.6	IB	fine sand	MG	68	Mperedens (48); Mthrix (36)	bd	bd	68
C	13H-2	111.1	28.2	IB	clay	MG	74	Mperedens (44)	bd	bd	74
D	1H-2	121.5	30.7	IB	clay	AOM	667	Mperedens (6); ANME-2 (13); ANME-3 (14); Mregulaceae (9); Mcell (3)	67	ANME-1-a, -d (43)	734
D	3H-2	141.8	35.5	IB	silty clay	SR	bd	bd	bd	bd	bd
D	5H-4	162.5	40.4	IB	fine sand	SR	22	bd^7^	bd	bd	22
C	16H-2	190.2	47.0	IB	clay	SR	6	bd^7^	6	ANME-1-d (45)	12
C	18H-3	249.3	61.0	II	clay	SR	6	Mperedens (38)	bd	bd	6
C	19H-3	258.8	63.3	II	clay	SR	bd	bd	bd	bd	bd
C	19H-5	262.9	64.3	II	clay	SR	21	Mperedens (33)	bd	bd	21

For each primer combination we include minimum copy numbers based on MPN-PCR and detected taxonomic groups based on clone libraries. For ANME-1, which included four distinct phylotypes (a-d), we show distributions of these phylotypes.

1 The code is as follows: 1H-2 = core 1, section 2, sampled by advanced piston core (H).

2 From Lever et al. (2006).

3 SR = sulfate reduction zone; AOM = anaerobic oxidation of methane zone; MG = methanogenesis zone.

4 Generated with mcrIRD primer pair; Abbreviations: Mperedens = Methanoperedenaceae, Mthrix = Methanothrix; Mregulaceae = Methanoregulaceae; Mcell = Methanocellaceae (names are to the highest taxonomic level identifiable).

5 Generated with ANME-1-mcrI primer pair.

6 Sum of minimum mcrA copy numbers obtained with both primer combinations.

7 Despite the presence of PCR products in the size of mcrA in these samples, attempts to sequence these PCR products were not successful.

**Figure 3:**
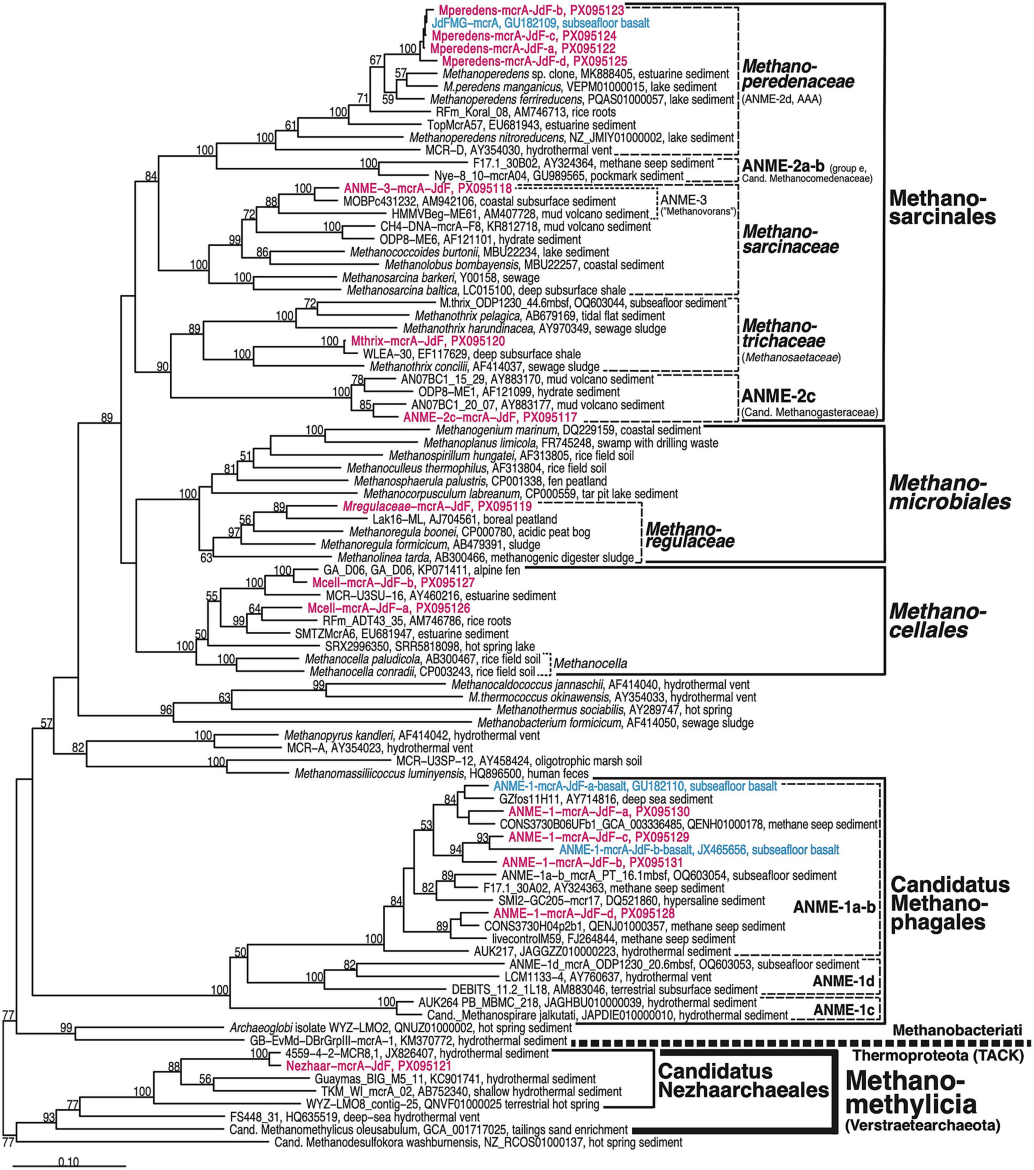
*Mcr*A phylogenetic tree. Representative sequences in magenta font are from U1301 sediments. Sequences in blue font are from U1301 basalt. Constructed in ARB Neighbor Joining using Jukes-Cantor correction. Bootstrap values (in %, 1,000 replications) are indicated for branching points with ≥50% bootstrap support.

**Figure 4:**
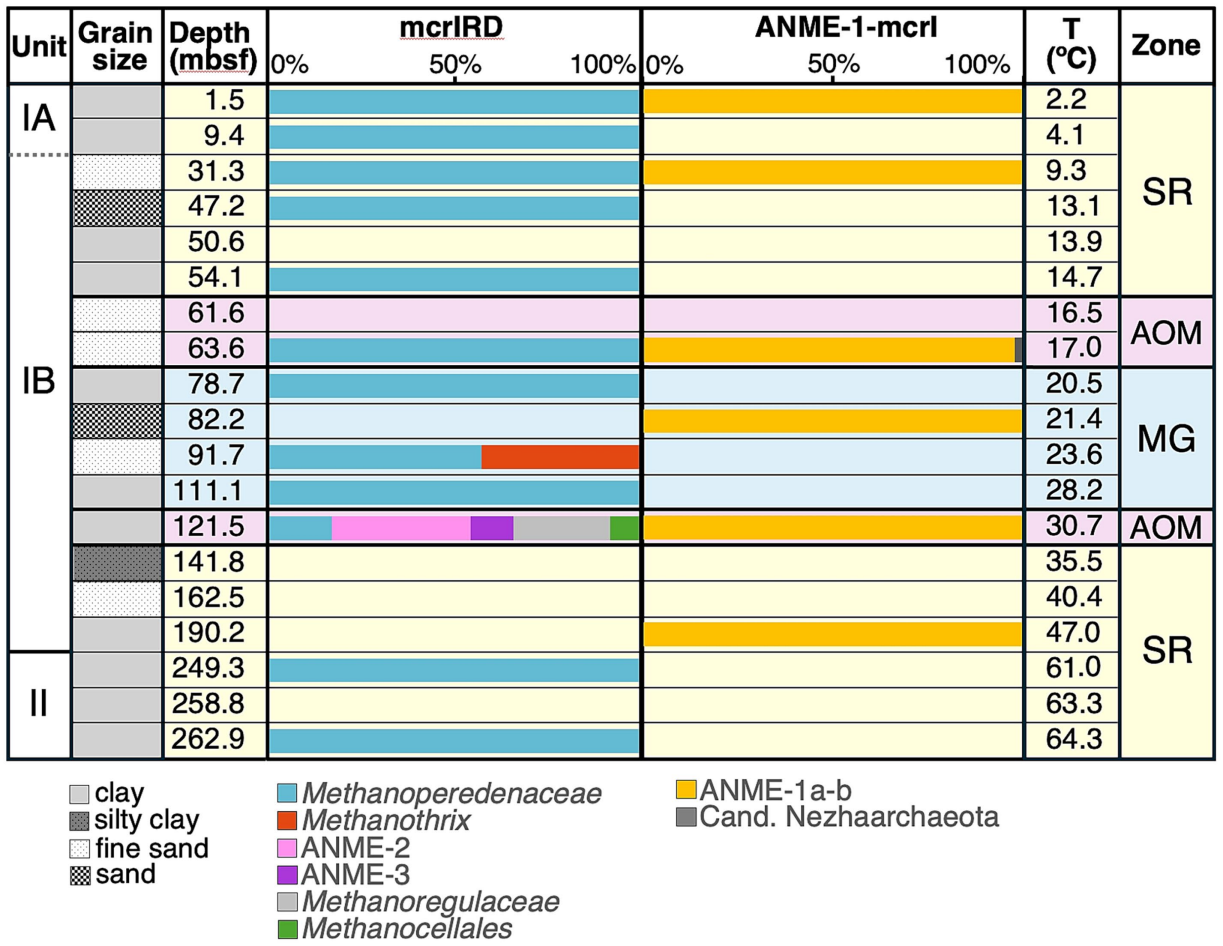
Bar chart illustrating distributions of *mcr*A phylogenetic clusters in relation to geological units, dominant grain size, sediment depth, primer pair, *in situ* temperature, and biogeochemical zone.

Sequences corresponding to *mcr*A fell into 12 different phylotypes that belong to 8 phylogenetic clusters (Figure 3). Of these, 7 phylotypes (6 phylogenetic clusters) were recovered using the mcrIRD primer pair, whereas 5 phylotypes (2 phylogenetic clusters) were obtained using the ANME-1-mcrI primer pair. The dominant cluster among mcrIRD reads belong to the family *Methanoperedenaceae* (order *Methanosarcinales*), members of which couple AOM to the reduction of nitrate, iron(III), and manganese(IV) (Ettwig et al., 2016; Leu et al., 2020). This family accounted for the majority of *mcr*A sequences in all samples except Core 1H-2 from the lower AOM zone (Table 1; Figure 4). Despite the wide range of *in situ* temperatures (2 to 64°C), lithologies (clay to sand), and biogeochemical zones (SR, AOM, MG), the four representative *mcr*A sequences of this group (Mperedens-mcrA-JdF-a, -b, -c, -d; Figure 3) all belong to the same phylotype. This phylotype includes a previously sequenced *mcr*A sequence (JdFMG-mcrA) from underlying subseafloor basalt of Hole U1301B (mean nucleotide distance±SD among sediment and basalt sequences: 1.1 ± 0.8%; Figure 3; Supplementary Figure 1A). Both phylogenetic analyses (Figure 3) and a Nucleotide Blast (blastn) search (Altschul et al., 1990) through the NCBI database indicate that the four sediment and one basalt *Methanoperedenaceae mcr*A sequences from Site U1301 are more similar to each other than they are to any other previously published *mcr*A sequences. In addition, the sequences from this study form a subcluster (100% bootstrap support) within the *Methanoperedenaceae* that includes two isolates that were previously shown to couple AOM to manganese and iron reduction [*Candidatus* (*Ca.*) Methanoperedens manganicus, *Ca.* Methanoperedens ferrireducens; Leu et al., 2020; Figure 3**]**.

After the *Methanoperedenaceae*, the ANME-1a-b group (candidate order Methanophagales) was the next most widely detected phylogenetic cluster (6/19 samples). This group, which was only detected using the ANME-1-mcrI primer pair, was found across a range of *in situ* temperatures (2.2 to 47.0°C) and lithologies (clay to sand). Despite being frequently linked to energy conservation by sulfate-dependent AOM (Knittel et al., 2019; Wang et al., 2021), ANME-1a-b *mcr*A was detected in SR, AOM, and MG zones (Table 1; Figure 4), in line with research suggesting that this group includes facultative methanogens (e.g., Beulig et al., 2019; Kevorkian et al., 2021; Deng et al., 2025). Different from *Methanoperedenaceae*, however, the four selected ANME-1 sequences (ANME-1-mcrA-JdF-a, -b, -c, -d) belong to distinct phylotypes (mean nucleotide distance±SD: 10.8 ± 2.9%; Supplementary Figure 1B), as well as different lineages within the ANME-1a-b branch of the *mcr*A tree (Figure 3). While ANME-mcrA-JdF-1-d was detected in 5/6 samples, the other phylotypes were each only detected in one (ANME-1-mcrA-JdF-b, -c) or two samples (ANME-1-mcrA-JdF-a) (Table 1). Notably, ANME-1-mcrA-JdF-a and -c had higher *mcr*A sequence similarities to previously sequenced ANME-1 phylotypes from underlying basalt than to ANME-1-mcrA-JdF-b or -d (Figure 3; Supplementary Figure 1B).

The remaining 6 *mcr*A clusters were limited to the AOM and MG zones. Four of these groups (ANME-2c (*Ca.* Methanogasteraceae), ANME-3 (*Ca.* Methanovorans) (both *Methanosarcinales*), *Methanocellales*, and *Methanoregulaceae* (*Methanomicrobiales*) were only detected in Core 1H-2 of Hole U1301D (121.5 mbsf; Table 1), which belongs to the lower AOM zone (Figure 1C). While ANME-2c and -3, which together account for approximately half of all reads in this layer, lack cultured members, past evidence has indicated both clusters to be methanotrophic via sulfate-dependent AOM (Boetius et al., 2000; Lösekann et al., 2007; Knittel et al., 2019). By contrast, all cultured members of *Methanocellales* (formerly Rice Cluster 1) and *Methanoregulaceae* perform methanogenesis via CO_2_ reduction using H_2_ or formate as electron sources (Sakai et al., 2008; Oren, 2014). An additional phylotype, belonging to the genus *Methanothrix* (family *Methanotrichaceae;* order *Methanosarcinales*) was detected in one sample within the MG zone, where this group accounted for nearly half of all *mcr*A reads (Table 1; Figure 4). While all members of this group were previously thought to be obligately aceticlastic methanogens (Whitman et al., 2014; Smith and Ingram-Smith, 2007), more recent studies indicate the capacity of some members to also perform methanogenic CO_2_ reduction via direct interspecies electron transfer (DIET) (Rotaru et al., 2014; Zhou et al., 2023). Lastly, while all *mcr*A sequences discussed so far belong to the phylum Methanobacteriota, we also detected a single sequence belonging to the candidate order Nezhaarchaeales within the Thermoproteota in the upper AOM zone (Core 8H-2; Table 1; Figure 4). This phylotype has a high sequence similarity to *mcr*A sequences that were previously found in hydrothermal sediments of Guaymas Basin (Figure 3). Based on genomic and *mcr*A expression data, Nezhaarchaeales are hydrogenotrophic methanogens that potentially generate methane via reduction of C1 compounds with H_2_ (Wang et al., 2023; Lynes et al., 2023; Qi et al., 2024).

## Discussion

Past efforts involving 16S rRNA gene-targeted amplicon (Labonté et al., 2017) and metagenome sequencing (Metze et al., 2023) were unsuccessful at detecting known methane-cycling archaea in subseafloor sediments of Site U1301 on the Juan de Fuca Ridge Flank. By targeting *mcr*A, a gene which has been found in all known methanogenic and anaerobic methane-oxidizing archaea (Friedrich, 2005; Wang et al., 2021), we were able to show that both groups are indeed present, explaining the methane concentration profile of the site, which indicates a deep zone of methane production that is flanked by zones of AOM and SR (Figures 2A,B). A similar outcome was previously obtained in biogenic methane-rich subseafloor sediments of the Peru Trench (ODP site 1,230). There also 16S rRNA gene and metagenomic surveys failed to detect methanogenic or methane-cycling archaea (Inagaki et al., 2006; Biddle et al., 2006, 2008), while an *mcr*A survey detected both groups over wide parts of the MG and AOM zones (Lever et al., 2023).

As pointed out before, the main reason for unsuccessful efforts of detecting methane-cycling archaea with universal assays is most likely the low *in situ* abundance of these organisms (Lever, 2013). At Site U1301, it is questionable if methane-cycling archaea reach populations above 10^3^ cells per cm^−3^, even in cores with peak *mcr*A copy numbers in the shallow SR (U1301C, Core 4H-5; ≥623 copies cm^−3^) and lower AOM zones (U1301D, Core 1H-2; 667 copies cm^−3^) (Table 1). Even in these cores, total cell counts were ~5 to 6 orders of magnitude higher (4H-5: 3.5 × 10^8^ cells cm^−3^; 1H-2: 7.9 × 10^7^ cells cm^−3^; Engelen et al., 2008). A similar ~10^5^-fold discrepancy is seen between *mcr*A copy numbers from deep sediment layers of this study (e.g., U1301C, 18H-3 and 19H-5; Table 1) and total prokaryotic 16S rRNA gene copies from corresponding sediment layers at nearby IODP Site 1362A (Labonté et al., 2017). If methane-cycling archaea indeed only account for ~0.001% of the total microbial populations, then this would help explain their only rare detection in sequencing assays with universal microbial targets. These low abundances are, nonetheless, remarkable given the crucial functions played by methanogens, as dominant terminal oxidizers of organic matter in MG zones, and of methane oxidizers, which prevent methane generated in MG zones from diffusing beyond the adjacent AOM zones.

The vertical abundance profile of methane-cycling archaea at U1301 is furthermore notable in that the highest *mcr*A copy numbers were not in the MG zone, but in the upper SR zone and lower AOM zone. The peak in the SR zone defies past indications that sulfate reducers outcompete methanogens in the presence of high sulfate concentrations but is consistent with the measured peak in potential methanogenesis rates in sulfate-reducing subseafloor sediment of the Peru Margin (Parkes et al., 2005). Interestingly also, the contribution of methane-cycling archaea to the total community does not change in the MG zone, where both *mcr*A copies and cell counts were ~1 order of magnitude below those in the upper SR zone (Table 1; Engelen et al., 2008). A relative (~5-fold) increase in the ratio of *mcr*A copies to total cell counts, that coincided with a ~ 10-fold increase in total *mcr*A copies, was only observed in the lower AOM zone (Table 1), suggesting localized growth stimulation by AOM. The absence of a clear growth stimulation of methane-cycling archaea in the MG zone of Site U1301 differs from other subseafloor sediments where total *mcr*A copy numbers were shown to increase (Deng et al., 2025) or only became detectable (Lever et al., 2023) in sulfate-depleted layers. We propose that net growth of methanogens in the MG zone of U1301 is either absent or masked by strong vertical variations in organic carbon contents (~0.1 to ~0.7 wt. %) and dominant organic carbon sources; the latter vary from phytoplankton-derived to “pre-aged” terrestrial organic matter (Fisher et al., 2005).

The zonation of *mcr*A phylotypes at U1301 indicates a strong geochemical determinant of methane-cycling archaeal community composition, with the highest diversity of *mcr*A in sediment layers where sulfate is depleted. This central role of sulfate, and potentially other high-energy electron acceptors (e.g., Fe(III), Mn(IV)), in driving methane-cycling archaeal community structure in marine subsurface sediments has been shown previously (e.g., Lever et al., 2023; Deng et al., 2025), and is perhaps even expected given the energetic disadvantages methanogens face compared to sulfate and metal reducing microorganisms (e.g., Hoehler et al., 1998). Similarly, the lack of a clear community zonation in relation to lithology and organic matter compositions may not be surprising. Though mineral interactions influence methanogenic growth in laboratory experiments (Xiao et al., 2022) and conductive minerals are involved in inter-species electron transfer (Rotaru et al., 2019; Meier et al., 2024), environmental zonations of methane-cycling archaea in relation to changing mineralogy or organic matter chemical compositions have not been demonstrated (e.g., Meier et al., 2024; Su et al., 2025). This could be because most methanogenic taxa feed on universal fermentation products, such as H_2_ or acetate, that are produced during the fermentative breakdown of all complex organic matter, independent of its organismal source (Schink and Stams, 2013; Conrad, 2020). Consequently, methanogenic energy substrates may not change significantly downcore, despite major variations in mineralogy, organic matter sources, and organic matter chemical compositions. Supporting this argument, research on lake sediments has shown only minimal changes in methanogenic communities across sediments with different organic matter sources and chemical compositions (Meier et al., 2024; Su et al., 2025).

Perhaps the most remarkable aspect about the methane-cycling archaeal community profile at U1301 is the lack of a clear temperature zonation. The same phylotypes of *Methanoperedenaceae*, and—if underlying basalt is included—ANME-1a-b, dominate over a temperature range of >60 °C (+2 to +64 °C) that spans the traditional psychrophilic, mesophilic, and thermophilic temperature ranges of microorganisms. To our knowledge, this surpasses the known temperature range of methanogens in the laboratory (record: 55 °C for a *Methanothermobacter thermoautotrophicus* strain; Wagner and Wiegel, 2008) and similar temperature ranges have only been reported for *Bacilli* isolates from terrestrial hot springs (Pandey et al., 2015). Instead, environmental surveys have commonly reported microbial, including methane-cycling archaeal, community shifts (zonations) across comparable temperature ranges (e.g., Dhillon et al., 2005; Lever and Teske, 2015; Lagostina et al., 2021). While long-term preservation of “fossil” DNA from dead microbial cells can lead to problematic inferences (Corinaldesi et al., 2011; Torti et al., 2015), fossil DNA does not explain the clear geochemical evidence for methane-cycling in U1301 sediment (Figure 2) and underlying basalt (Lever et al., 2013). In addition, at temperatures >60 °C, active DNA repair is essential for DNA to remain detectable over periods of thousands of years given the rapid chemical degradation of DNA via depurination (average nucleotide half-life at 60 °C: <100 years; Appendix Figure A6 in Lever et al., 2015). Physical isolation from colonization by ecophysiologically more “fit” thermophilic methane-cycling archaea also does not explain the dominance of single phylotypes. The one phylotype of *Methanoperedenaceae* and two of the four phylotypes of ANME-1a-b were also dominant in underlying basalt, which has active circulation of fluids that are of recent seawater origin and are known to transport microbial cells (Huber et al., 2005; Jungbluth et al., 2016). Potential source environments of thermophilic methane-cycling archaea are located further west at the Juan de Fuca Ridge, the seafloor spreading center that originally produced the basaltic basement at U1301. The Juan de Fuca Ridge is a well-known methanogenic habitat (*Methanocaldococcus* spp.; ver Eecke ver Eecke et al., 2012; Anderson et al., 2013) and may have been the source of thermophilic *Methanothermococci* and *Archaeoglobi* that were found in hot fluid-exposed, corroded borehole pipe ~1 km south of U1301 at ODP Site 1026B (Nakagawa et al., 2006; Steinsbu et al., 2010).

### Methanogenic substrates and dominant methanogenic metabolism

The dominance, and even sole occurrence, of two clusters of methane-cycling archaea in the SR zones and other parts of the U1301 sediment column raises questions regarding the ecological niches of these clusters, including their energy sources and inter-relationships. Based on previous research on the underlying basalt at U1301, where the same two clusters also dominate, a complete methane cycle with *in situ* methane production and consumption was proposed (Lever et al., 2013). This explanation is also plausible for the SR zones of U1301 sediments, where a narrow, low micromolar methane concentration range (1–3 μM; Fisher et al., 2005) implies tight regulation of *in situ* methane concentrations. Measurable AOM rates throughout the sediment column of U1301 further support this notion of a “cryptic” methane cycle (Engelen et al., 2008), which was also proposed for coastal and shelf sediments (Krause et al., 2025; Deng et al., 2025).

**Figure 5:**
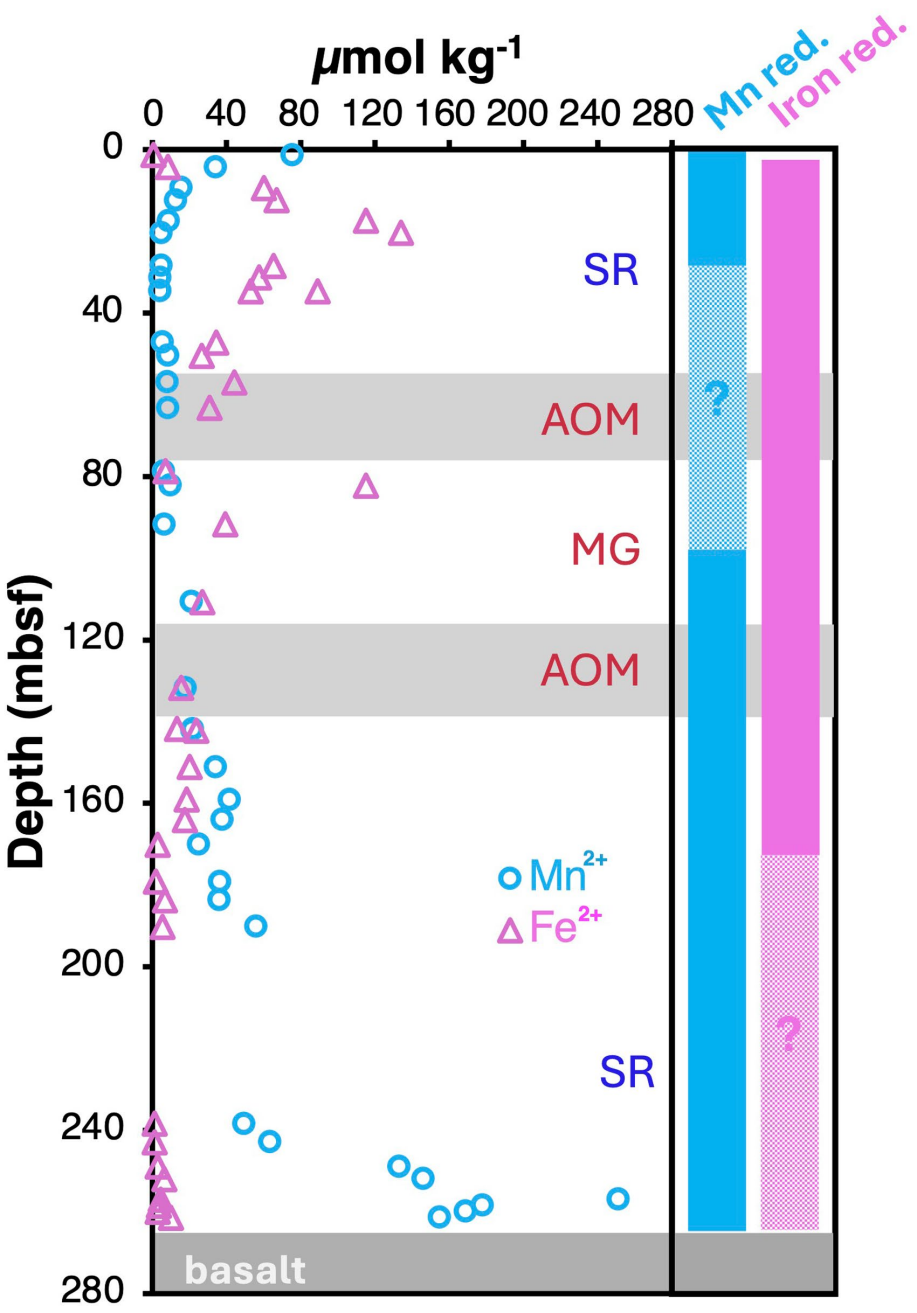
Depth profiles of measured Mn^2+^ and Fe^2+^ concentrations (data from Fisher et al., 2005). Vertical bars on far right illustrate approximate zones of Mn(IV) reduction (solid blue) and Fe(III) reduction (solid magenta). Hatched areas indicate sediment intervals in which the presence of Mn(IV) or Fe(III) reduction is unclear based on measured Mn^2+^ and Fe^2+^ concentrations.

Yet, the roles of the methanogenic and methanotrophic partner organisms may differ from those originally proposed by Lever et al. (2013). The basalt phylotype (JdFMG-mcrA; Figure 3) was previously thought to be methanogenic based on the enrichment of a closely related phylotype in wetland sediments under methanogenic conditions (Zhang et al., 2008). Yet, members of this phylogenetic cluster have since been isolated and shown to perform AOM using nitrate, Fe(III), and Mn(IV), but not sulfate, as electron acceptors (Haroon et al., 2013; Ettwig et al., 2016; Leu et al., 2020). These pure culture isolates led to the classification of this cluster, which was previously referred to as ANME-2d (Hallam et al., 2003) or AOM-associated group (AAA; Knittel and Boetius, 2009), as the family *Methanoperedenaceae* (Haroon et al., 2013). We therefore suggest that the *Methanoperedenaceae* detected at U1301 are performing AOM in both sedimentary and basaltic habitats. Given that significant nitrate concentrations are unlikely in these deeply anoxic environments, whereas terrestrial sediment layers and underlying basalt are rich in iron and manganese (Fisher et al., 2005), AOM coupled to Fe(III) and Mn(IV) reduction is the most plausible source of energy. Support for a major contribution of sulfate-independent AOM comes, at least locally, from the fact that sulfate concentrations were at background values throughout the upper AOM zone (Figure 2). In addition, Mn^2+^ and Fe^2+^ concentrations were measurable throughout most of the sediment column, suggesting active metal cycling with overlapping zones of iron reduction, manganese reduction, SR, AOM, and MG (Figure 5). Notably, the Fe(III)- and Mn(IV)-reducing *Ca.* Methanoperedens ferrireducens and *Ca.* Methanoperedens manganicus form a distinct phylogenetic subcluster with the *Methanoperedenaceae* detected at U1301 (Figure 3), suggesting close genetic relatedness.

If *Methanoperedenaceae* are indeed oxidizing methane within the SR zones of U1301, then this inevitably raises questions about the sources of this methane. Given that thermochemical methane production is thought to only become significant at temperatures >80 °C (Horsfield et al., 2006), a microbial source of methane is likely. While bacteria produce methane via methylphosphonate cleavage under phosphorus limitation in marine water columns (Repeta et al., 2016; von Arx et al., 2023), this process has not been reported for sediments. Micromolar phosphate concentrations and low phosphatase activities in the upper 160 mbsf of U1301, which includes the entire upper SR zone and parts of the lower SR zone, moreover, argue against P limitation (Engelen et al., 2008). Instead, ‘canonical methanogenesis’ by methanogenic archaea is the most likely source of methane. Members of ANME-1a-b, which have never been isolated into pure culture, were originally thought to exclusively perform AOM (e.g., Orphan et al., 2002; Lloyd et al., 2006). Yet, stable isotopic analyses, radiotracer incubations, gene expression profiles, and environmental distributions far into MG zones have since indicated that some ANME-1a-b are capable of a methanogenic lifestyle (e.g., Alperin and Hoehler, 2009; Lloyd et al., 2011; Beulig et al., 2019; Deng et al., 2025). Based on the observed distributions, we propose that the ANME-1a-b of SR zones in U1301 sediments and basalt are primarily acting as methanogens. The methane produced is subsequently consumed by widely co-occurring *Methanoperedenaceae*, leading to a cryptic methane cycle with tight coupling of MG and AOM.

The remaining methane-cycling archaeal phylotypes were only detected in single samples, making environmental interpretations difficult (Figure 4). We will refrain from further discussing the detection of a single sequence of Nezhaarchaeales, apart from pointing out that this group was previously mainly found in terrestrial hot spring environments (Wang et al., 2023; Lynes et al., 2023; Qi et al., 2024) and hydrothermal marine environments (ver Eecke et al., 2012; Nunoura et al., 2013). By contrast, the sole detection of *Methanothrix* within the MG zone is consistent with prior data suggesting that this group of aceticlastic methanogens (Whitman et al., 2014), some members of which can also perform electrotrophic CO_2_ reduction (Rotaru et al., 2014; Gao and Lu, 2021), are rare outside of MG zones in marine sediments (Bae et al., 2015; Lever et al., 2023; Deng et al., 2025).

The detection of multiple putative methanotrophs (ANME-2c, ANME-3, *Methanoperedenaceae*) along with two groups of CO_2_-reducing methanogens (*Methanoregulaceae*, *Methanocellales*) in the lower AOM zone suggests that in addition to AOM, there may also be methane production in this layer. Based on the overlapping sulfate and methane concentration gradients and clear indications of sulfate-reducing activity in the lower AOM zone (Figure 2), and the significant concentrations of Fe^2+^ and Mn^2+^, we propose the co-occurrence of sulfate- and metal-dependent AOM. Herein *Methanoperedenaceae* directly couple AOM to Fe(III) and Mn(IV) reduction, whereas ANME-2c and ANME-3, which dominate *mcr*A read percentages in this layer, most likely perform sulfate-dependent AOM in syntrophic associations with sulfate-reducing bacteria (McGlynn et al., 2015; Woods et al., 2025). More difficult to explain is the detection of putatively CO_2_-reducing *Methanoregulaceae* and *Methanocellales*, though this is consistent with previous detections of significant read percentages of putatively CO_2_-reducing *Methanomicrobiaceae* in AOM zones (Dalcin Martins et al., 2024; Deng et al., 2025). This is because thermodynamically a reaction cannot be favorable in the forward and reverse direction within the same chemical environment. While chemical micro-environments (microniches) with distinct chemical conditions exist in surface environments with high metabolic activities (Anderson and Meadows, 1978; Bartosiewicz et al., 2023), the high substrate turnover rates that are required to counteract dissipation of microgradients by molecular diffusion seem unlikely for low-activity subseafloor environments. More plausible explanations are that these putative CO_2_-reducing methanogens are reversing their metabolism and performing AOM (Kevorkian et al., 2022), or that the CO_2_ reduction and AOM reactions are not strict reversals of each other. For instance, CO_2_ reduction could involve soluble electron carriers (e.g., H_2_, formate), while AOM by ANME-2c and ANME-3 could proceed by direct interspecies electron transfer to syntrophic partner organisms.

### Co-occurrence of competing terminal oxidation reactions

If metal reduction continues throughout the sediment column and is a major driver of AOM at U1301, then why are MG and SR, which likely produce lower *in situ* energy yields than Mn(IV) or Fe(III) reduction, still occurring? One explanation is that low-reactivity metal oxide phases impose kinetic limitations on the rates of iron and manganese reduction (Roden, 2004), causing these metal oxides to escape microbial reduction in near-surface sediments and to be buried to deeper layers (Lovley and Phillips, 1986; Canfield, 1989). If the rate of electron donor supply by organic matter degradation subsequently exceeds the rates at which Fe(III) and Mn(IV) oxides become available for microbial reduction, then the resulting oversupply of electron donors could explain the coexistence of energetically less favorable SR, MG (*this study*), and even acetogenesis reactions (Lever et al., 2010). Alternatively, most of the Fe(III) and Mn(IV) reduction in U1301 sediments could be abiotic rather than microbial. Abiotic Fe(III) and Mn(IV) reduction via hydrogen sulfide oxidation or elemental sulfur disproportionation has been proposed for other subseafloor sedimentary systems (e.g., Riedinger et al., 2014; Torres et al., 2015), and for the underlying basalt at U1301 (Lever et al., 2013). These reactions can, in principle, explain the elevated Fe^2+^ concentrations in the upper SR zone, as well as the elevated Mn^2+^ concentrations from 0–20 mbsf and close to the basement (≥240 mbsf; Figure 5). Yet, abiotic reactions do not plausibly explain the elevated Fe^2+^ concentrations in the sulfate-depleted MG zone, where the production of hydrogen sulfide or elemental sulfur is expectedly minimal. In addition, the widespread detection of *Methanoperedenaceae* that couple the oxidation of methane to the reduction of Mn(IV) or Fe(III) argues against a solely abiotic source of dissolved Fe^2+^ or Mn^2+^.

This leaves the question, why MG likely occurs in the presence of SR. The detection of putative methanogenic activity in SR zones has traditionally been explained with the use of non-competitive substrates, e.g., methanol, methyl sulfides, methyl amines, by methanogens. Yet, the typical methyl-disproportionating taxa within the *Methanosarcinaceae* that are widespread in SR zones of marine sediments (Oremland and Boone, 1994; Xiao et al., 2018; Zhuang et al., 2018; Deng et al., 2025) were not detected in sediments of U1301. If ANME-1a-b are indeed the main producers of methane in the U1301 sediment column and using canonical methanogenic archaeal energy substrates, then further research will need to elucidate whether this methane is produced (1) from non-competitive substrates (Bertram et al., 2013), (2) via CO_2_ reduction using electrons directly supplied from (a) syntrophic partner organisms (e.g., Rotaru et al., 2014) or (b) abiotically oxidized minerals (e.g., olivine; Lever et al., 2013), or (3) whether competitive substrates, such as H_2_ or acetate, are after all present in sufficiently high concentrations to support methanogenesis.

## Conclusion

Our combined data set on the sediment column of IODP Site U1301 illustrates the importance of integrating geochemical and microbiological analyses to understand subsurface biogeochemical processes. Based on measured concentration profiles of methane and sulfate it would be plausible to infer that AOM and MG follow standard scenarios, wherein AOM is largely coupled to SR and MG only becomes quantitatively important in deeper layers where electron acceptors, such as Mn(IV), Fe(III), or sulfate, that support respiration reactions with higher energy yields are depleted. Yet, adding microbiological data based on *mcr*A copy numbers and phylogenetic analyses changes the interpretation, namely suggesting that MG and AOM occur simultaneously throughout major parts of the sediment column, including SR, AOM, and MG zones. In addition, the apparent disconnect between SR and AOM in the upper AOM zone, and the pervasive dominance of Fe(III)- and Mn(IV)-reducing ANMEs (*Methanoperedenaceae*), suggest that a major part of AOM in U1301 sediments does not proceed through sulfate as the electron acceptor. The microbiological data set is obviously limited to a small sequencing depth (mcrIRD: 500 sequences; ANME-mcrI: 251 sequences) and sample size (n = 19). Yet, the results, in particular the pervasive dominance of *Methanoperedenaceae* and ANME-1a-b, which was also previously reported for underlying basalt (Lever et al., 2013), are consistent. Why only two groups of methane-cycling archaea dominate over the wide range of temperature, redox conditions, and lithologies at U1301 remains enigmatic. We suggest that metabolic versatility, lack of dependence on specific syntrophic partner taxa, and broad temperature ranges are key characteristics that explain the widespread dominance of *Methanoperedenaceae* and ANME-1a-b.

The *mcr*A copy number peak in the upper SR zone, and absence of elevated relative abundances of methane-cycling archaea in the MG zone, contrasts with previous studies that indicated increases in relative and absolute abundances of methane-cycling archaea in sulfate-depleted subseafloor sediments (Lever et al., 2023; Deng et al., 2025). In the absence of a larger subseafloor dataset further interpretations are challenging. As a working hypothesis for future research, we propose that elevated metal contents in sediments with high terrestrial or hydrothermal sedimentary inputs facilitate the co-existence of MG with other, more energy-yielding respiration reactions. Herein conductive metal surfaces may facilitate the direct transfer of electrons between methanogens and potential partner organisms and enable the coexistence of competing metabolic pathways. The direct transfer of electrons may be crucial, as it bypasses soluble interspecies electron carriers, such as H_2_, which microorganisms can draw down to minimum concentrations that only support growth of the most energetically favorable terminal oxidation reaction (Hoehler et al., 1998). Direct electron transfer via metals may also play a role in other sediments with lower iron or manganese contents, thus explaining the widespread occurrence of low abundances of CO_2_ reducing methanogens in SR zones (e.g., Deng et al., 2025; Wallenius et al., 2025). Yet, we speculate that the importance of direct interspecies electron transfer increases significantly in metal-rich environments, such as IODP Site U1301 on the eastern flank of the Juan de Fuca Ridge with its high terrestrial and hydrothermal sediment contributions and volcanic ash deposits.

## Data Availability

All geochemical data are publicly available in Fisher et al. (2005). All mcrA sequences are publicly accessible through the National Center for Biotechnology (NCBI) homepage (nucleotide accession numbers PX095117-PX095131).
