# Supplementary figures and images for: Distribution of methane-cycling archaea in buried ridge flank sediment: community zonation, activity, and potential environmental drivers
Source: Front Microbiol. 2025 Nov 24;16:1710699. doi: 10.3389/fmicb.2025.1710699 (PMC12683337; doi:10.3389/fmicb.2025.1710699)

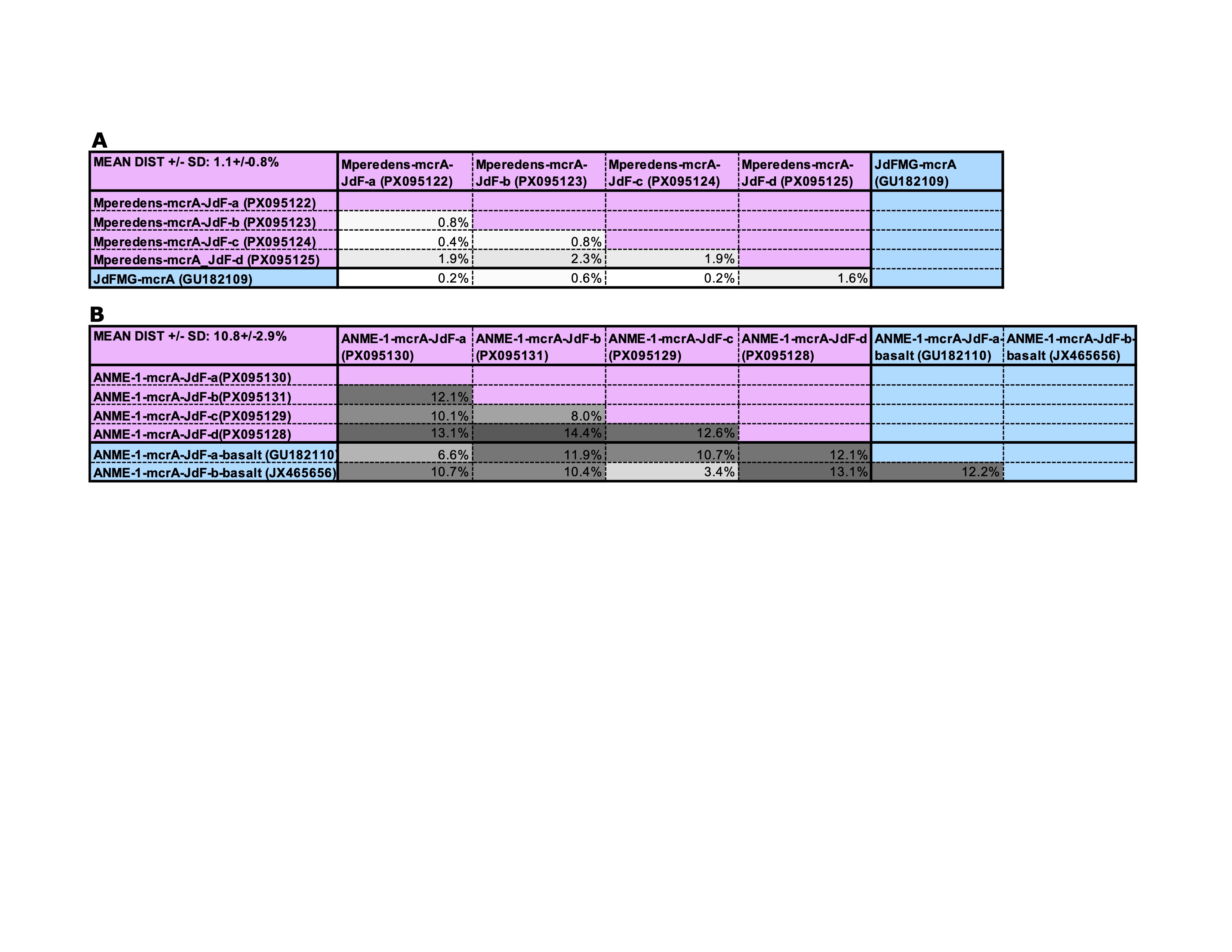

Supplement: Supplementary file 1 [file Image_1.JPEG]
